# Readability of Professional Medical Content on Acute Appendicitis: A Comparative Cross-Sectional Study of ChatGPT and UpToDate

**DOI:** 10.7759/cureus.95898

**Published:** 2025-11-01

**Authors:** Maira Jalil, Saow Renn Ding, Nikita M Talpallikar, Ishita Bhatnagar, Shree V Umashankar, Jocelyn N Wensel

**Affiliations:** 1 Internal Medicine, University of Debrecen, Faculty of Medicine, Debrecen, HUN; 2 Internal Medicine, St. George's University School of Medicine, University Centre Grenada, St. George’s, GRD; 3 Internal Medicine, D.Y. Patil Medical College, Kolhapur, IND; 4 Internal Medicine, Gujarat Medical Education and Research Society (GMERS) Medical College and Hospital, Himatnagar, IND; 5 Internal Medicine, St. Martinus University, Faculty of Medicine, Willemstad, CUW; 6 General Medicine, International University of the Health Sciences, Basseterre, KNA

**Keywords:** acute appendicitis, chatgpt, complex words, grade level, readability, smog index

## Abstract

Introduction

Acute appendicitis is one of the most common diseases occurring due to inflammation of the vermiform appendix, which requires surgical intervention. With the advent of standardized artificial intelligence (AI) tools such as ChatGPT (OpenAI, San Francisco, CA), AI-based search engines have emerged as a secondary means for patients to educate themselves about their health. The readability of each response is important for concept understanding as well as the impact of novel therapeutics.

Aims

This study aims to evaluate and compare the readability of medical information on acute appendicitis generated by an AI language model and UpToDate (Wolters Kluwer Health, Waltham, MA) using established readability metrics.

Methodology

A comparative cross-sectional study was conducted to evaluate the readability of six ChatGPT-4o and six UpToDate responses on acute appendicitis. Readability parameters were assessed using WebFX (WebFX®, Harrisburg, PA), and differences between sources were analyzed using the Mann-Whitney U test in IBM SPSS Statistics software, version 25 (IBM Corp., Armonk, NY) and R (v4.3.2, The R Core Team, R Foundation for Statistical Computing, Vienna, Austria).

Results

UpToDate had a higher word count and higher words per sentence than ChatGPT (both p < 0.05). ChatGPT had a lower absolute difficult-word count (p = 0.002) but a higher difficult-word percentage (p = 0.002). Differences in Flesch Reading Ease (FRE), Flesch-Kincaid Grade Level (FKGL), Simple Measure of Gobbledygook (SMOG), and sentence count were not statistically significant (all *p* > 0.05).

Conclusions

ChatGPT produced more concise content compared to UpToDate, but its higher proportion of difficult words may limit comprehension, highlighting the need to balance brevity with readability in AI-generated medical information.

## Introduction

Acute appendicitis is the most common abdominal emergency worldwide, but its causes are not well understood, making a confident diagnosis before surgery challenging. Clinical assessment, including the Alvarado scale, is the mainstay of diagnosis, including biomarkers and imaging [1]. Although with a low mortality rate and an annual incidence of 96.5 to 100 cases per 100,000 adults, postoperative complications are common [1,2]. Acute appendicitis is caused by inflammation of the appendix, often due to a fecolith, infection, or idiopathic causes. Surgery is the mainstay of treatment, though non-surgical (conservative) methods are becoming more common. While most cases have a favorable outcome, serious complications like appendiceal abscess, peritonitis, sepsis, rupture, or even death can occur in rare cases [3].

Patients are likely to use online patient educational materials (OPEMs) to gain more information about acute appendicitis, which should be at the readability level of or below the health literacy level of the public, over the sixth-grade level recommended by the American Medical Association. Generative artificial intelligence (AI) chatbots have recently risen as potential sources of targeted online medical information for patients and caregivers while making medical decisions or understanding the disease. Existing online patient-oriented medical information has repeatedly been shown to be of variable quality and difficult readability [4]. To acknowledge the difference between how readable and accessible medical information is when generated by AI tools compared to a standard clinical reference, this study uses a grade-level readability score calculated using standard readability metrics like Flesch Reading Ease (FRE), Flesch-Kincaid Grade Level (FKGL), Simple Measure of Gobbledygook (SMOG) Index, and complex words. Lack of access to readable OPEMs may disproportionately affect patients with low health literacy [5-8]. Therefore, ensuring that online content is understandable and broadly available to mass audiences is a necessary component of increasing the impact of novel therapeutics and recommendations [9].

The aim of this study is to analyze and compare the content readability of medical content related to the diagnosis and management of acute appendicitis when generated by an AI language model, ChatGPT (OpenAI, San Francisco, CA), versus UpToDate (Wolters Kluwer Health, Waltham, MA), a widely used evidence-based medical resource. The comparison utilizes established readability metrics, including word and sentence counts, percentage of complex words, FRE, FKGL, and SMOG Index, to evaluate whether the content is written at a level appropriate for the average reader.

## Materials and methods

Study design and setting

We conducted a comparative cross-sectional study from June 15 to June 22, 2025, to analyze and compare the readability of medical information on acute appendicitis generated by ChatGPT-GPT-4o [10] and retrieved from UpToDate (clinical reference database) [11]. As the study did not involve human participants, patient data, or medical interventions, approval from an Institutional Ethics Committee was not required.

Data sources and search strategy

For ChatGPT, six standardized prompts were used to generate responses, corresponding to pediatric and adult acute appendicitis: “Acute appendicitis in children-Diagnostic imaging,” [12] “Acute appendicitis in children-Clinical manifestation and diagnosis,” [13] “Acute appendicitis in children-Management,” [14] “Acute appendicitis in adults-Diagnostic evaluation,” [15] “Acute appendicitis in adults-Clinical manifestation and differential diagnosis,” [16] and “Management of acute appendicitis in adults” [17]. Each prompt was entered verbatim into ChatGPT, and the full-text output was collected for analysis.

Parallel content was retrieved from UpToDate by searching with the following terms: “Diagnostic imaging in pediatric acute appendicitis,” “Clinical features and diagnosis of pediatric acute appendicitis,” “Management of pediatric acute appendicitis,” “Evaluation of suspected acute appendicitis in adults,” “Clinical features and differential diagnosis of acute appendicitis in adults,” and “Management of acute appendicitis in adults.” The corresponding UpToDate chapters were reviewed, and patient education-oriented sections were extracted verbatim for analysis.

Study variables and topic representation

The six prompts and search terms defined the six “topics”. Topics 1 through 3 referred to pediatric appendicitis, covering diagnostic imaging, diagnosis, and management, whereas Topics 4 through 6 addressed adult appendicitis, focusing on diagnostic evaluation, differential diagnosis, and management. Each topic pair (ChatGPT versus UpToDate) was analyzed independently for readability.

Readability analysis

All extracted texts were converted to plain text format and analyzed using the WebFX Readability Test Tool (WebFX®, Harrisburg, PA) [18]. The following metrics were calculated: FRE, FKGL, SMOG Index, word count, sentence count, words per sentence, difficult word count, and difficult word percentage. FRE scores range from 0 to 100, with higher values indicating easier readability; a score of 90-100 corresponds to an 11-year-old reading level, 60-70 to 13-15 years, and below 30 to college level. FKGL represents the U.S. school grade needed for comprehension; for example, a score of 6.0 corresponds to sixth grade. The SMOG Index estimates the years of education required to understand a passage, with a score of 7 indicating seventh grade and 13 indicating freshman college level [5-8]. Difficult words were defined as those with three or more syllables, excluding proper nouns, compound words, and familiar suffixes.

Statistical analysis

All data were analyzed using IBM SPSS Statistics software, version 25 (IBM Corp., Armonk, NY) and R (v4.3.2, The R Core Team, R Foundation for Statistical Computing, Vienna, Austria). Because the distributions were non-parametric, the Mann-Whitney U test was used to compare continuous variables, including word count, sentence count, words per sentence, FRE, FKGL, SMOG, difficult word count, and difficult word percentage, between ChatGPT and UpToDate. Two-tailed p-values <0.05 were considered statistically significant.

## Results

Statistically significant differences have been noted between the median word count, word/sentence count, difficult word count, and difficult word percentage as generated by the two AI tools. These have been noted in Table 1.

**Table 1:** Characteristics of the responses generated by UpToDate and ChatGPT +Mann–Whitney’s U Test. P-values <0.05 are considered statistically significant. * Indicates a significant P-value/P-value < 0.05.

	Median (IQR)		U Statistic	P-value^+^
	UpToDate	ChatGPT		
Word Count	1495.5 (1174.0 – 5352.0)	549.5 (263.0 – 658.2)	0	0.002*
Sentence Count	66.0 (52.0 – 279.5)	45.5 (18.8 – 87.5)	10	0.223
Word/Sentence Count	20.7 (19.2 – 23.7)	12.1 (7.8 – 16.6)	3	0.015*
Flesch Reading Ease (FRE)	12.1 (8.2 – 16.7)	14.4 (10.6 – 26.8)	12	0.394
Flesch-Kincaid Grade Level (FKGL)	16.8 (16.2 – 17.6)	15.1 (11.4 – 17.6)	12.5	0.420
Simple Measure of Gobbledygook (SMOG) Index	14.2 (13.6 – 15.0)	11.8 (9.0 – 14.8)	9	0.180
Difficult Word Count	443.0 (330.8 – 1515.0)	171.0 (106.2 – 217.8)	0	0.002*
Difficult Word Percentage	29.2 (27.8 – 29.7)	32.7 (31.2 – 34.4)	0	0.002*

The results demonstrate differences in several metrics. UpToDate responses had a significantly higher median word count compared to ChatGPT (P = 0.002). Similarly, the word-to-sentence ratio was also significantly greater for UpToDate compared to ChatGPT (P = 0.015). The number of difficult words used was higher for UpToDate versus ChatGPT (P = 0.002). Additionally, the percentage of difficult words used was lower in UpToDate (P = 0.002).

However, there were no statistically significant differences between the two sources in terms of readability scores. The FRE scores are not statistically significant (P = 0.394). Similarly, no significant differences were found in the FKGL (P = 0.420), SMOG Index (P = 0.180), or the sentence count (P = 0.223).

**Figure 1:**
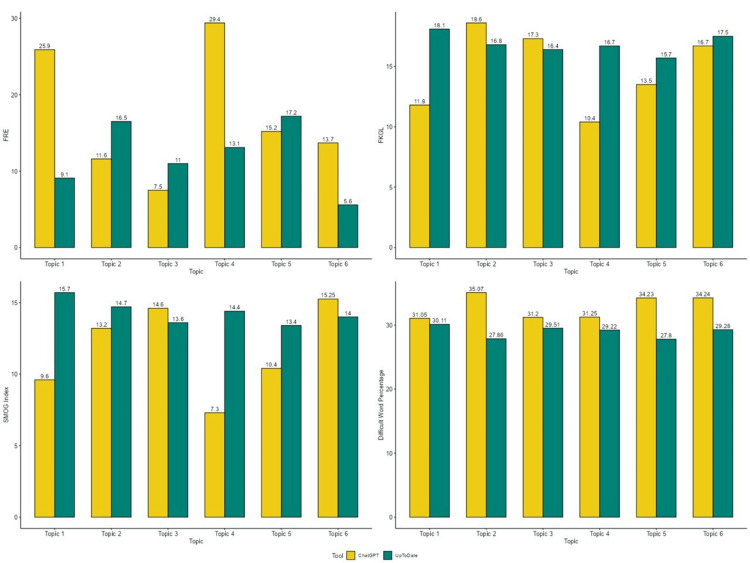
A graphical representation of the comparison between FRE, FKGL, SMOG Index, and difficult word percentage for the patient education guide generated by UpToDate and ChatGPT Topic 1: Diagnostic imaging in pediatric acute appendicitis; Topic 2: Clinical manifestation and diagnosis of pediatric acute appendicitis; Topic 3: Management of pediatric acute appendicitis; Topic 4: Diagnostic evaluation of acute appendicitis in adults; Topic 5: Clinical manifestation and differential diagnosis of acute appendicitis in adults; Topic 6: Management of acute appendicitis in adults FRE: Flesch Reading Ease; FKGL: Flesch-Kincaid Grade Level; SMOG: Simple Measure of Gobbledygook

Figure 1 shows a graphical representation of the comparison between FRE, FKGL, SMOG Index, and difficult word percentage for the patient education guide generated by UpToDate and ChatGPT. The findings indicate that ChatGPT generally produces content that is easier to read compared to UpToDate. In terms of FRE, ChatGPT typically scored higher or similarly across all topics, suggesting better readability, although Topics 2, 3, and 5 were an exception, where both were difficult to read, but UpToDate scored slightly better. There was a huge difference in FRE between ChatGPT and UpToDate in Topic 1 (ChatGPT 25.9 vs. UpToDate 9.1) and Topic 4 (ChatGPT 29.4 vs. UpToDate 13.1). For the FKGL, ChatGPT consistently scored lower, meaning it uses simpler language that requires a lower educational level to understand. Notable differences appeared in Topic 4 (ChatGPT 10.4 vs. UpToDate 16.7) and Topic 1 (ChatGPT 11.8 vs. UpToDate 18.1), indicating significantly easier comprehension with ChatGPT responses. The SMOG index also favored ChatGPT, with slightly lower scores in most topics, suggesting simpler vocabulary and sentence structures, except in Topic 3 and Topic 6, where ChatGPT scored marginally higher (14.8 vs. 13.6 and 15.25 vs. 14). Lastly, difficult word percentage was consistently higher in ChatGPT responses, with the most significant gap observed in Topic 2 (ChatGPT 35.067% vs. UpToDate 27.86%). Overall, while UpToDate tended to produce longer and more complex content, ChatGPT offered more accessible and easier-to-understand responses.

## Discussion

AI tools are increasingly utilized in medical education to generate accessible, structured clinical summaries. Large language models like ChatGPT offer point-of-care information, enabling clinicians to retrieve content efficiently, which is especially valuable in urgent scenarios such as acute appendicitis. These tools support just-in-time learning, reducing reliance on textbooks and providing on-demand insights for early-career professionals and trainees [19, 20]. In this study, ChatGPT produced compressed but informative content, potentially serving as a practical tool for clinical recall. However, the higher proportion of complex vocabulary suggests the need for editorial refinement before clinical use.

Readability scores, including the FRE, FKGL, and SMOG Index, evaluate how easily content can be understood. Higher FRE values indicate greater ease of reading, while lower FKGL and SMOG scores suggest content that is more accessible to broader audiences. In clinical settings, particularly with high-pressure diagnoses like appendicitis, clear and readable content supports quicker information absorption and decision-making [21]. In this study, ChatGPT and UpToDate both generated content exceeding the recommended readability thresholds for patient education, but are still deemed suitable for medical professionals. ChatGPT demonstrated slightly more favorable readability metrics, suggesting a potential advantage in fast-paced environments.

The findings of this study are consistent with prior research on AI in clinical education. Kung et al. (2023) reported that ChatGPT performed competently on standardized medical exams, showcasing its utility as a supplementary learning aid [22]. Patel and Lam (2023) found that ChatGPT produced discharge summaries that were linguistically simplified but sometimes lacked detailed context [23]. Similarly, Jeblick et al. (2022) observed that ChatGPT-generated radiology reports were well-structured but varied in complexity based on input prompts [24]. These parallels highlight a shared trend across AI platforms: outputs are often coherent and syntactically sound but may require fine-tuning to optimize clinical relevance.

However, not all studies align with these results. Shen et al. (2023) and Salvagno et al. (2023) found that ChatGPT's content was sometimes overly generic, lacking depth, or unsuitable for nuanced clinical topics like oncology or ethics [25, 26]. In contrast, this study found that ChatGPT’s appendicitis content was specific and information-rich, albeit dense in terminology. These discrepancies could stem from differences in prompt structure, clinical topic, or model version, and underscore the need for context-specific evaluation of AI outputs.

Limitations

This study evaluated only one AI tool (ChatGPT) and one clinical condition (acute appendicitis), which limits the generalizability of the findings to other diseases or platforms. The version of ChatGPT used may not incorporate the latest evidence or guidelines, especially in fast-evolving fields. The analysis focused solely on linguistic and readability metrics without assessing the clinical accuracy, evidence alignment, or educational value of the content through expert review. Additionally, patient comprehension was not evaluated, despite the relevance of readability in both clinician- and patient-facing materials. Future research should examine multiple AI tools across various specialties, incorporating expert feedback and real-world usability assessments to better define the role of generative AI in medical education.

## Conclusions

This study compared the readability of professional medical content on acute appendicitis generated by ChatGPT and UpToDate. ChatGPT produced shorter responses with lower word and sentence complexity, while UpToDate provided more detailed but lengthier text. Although FRE, FKGL, and SMOG Index scores did not differ significantly, ChatGPT’s concise structure may enhance accessibility for readers. However, its higher proportion of difficult words highlights the need to balance brevity with clarity when using AI-generated medical information.
